# Changes in the Growth Hormone-IGF-I Axis in Non-obese Diabetic Mice

**DOI:** 10.1155/EDR.2000.9

**Published:** 2000

**Authors:** Daniel Landau, Yael Segev, Rina Eshet, Allan Flyvbjerg, Moshe Phillip

**Affiliations:** 1 Molecular Endocrinology Laboratory Soroka Medical Center Ben Gurion University of the Negev Beer Sheva Israel; 2 Felsenstein Medical Research Center Institute for Endocrinology and Diabetes Schneider Children's Medical Center Israel; 3 Institute of Experimental Clinical Research Aarhus Kommunehospital Aarhus C. Denmark; 4 Department of Pediatrics Soroka Medical Center P. O. Box 151 Beer Sheva 84101 Israel

## Abstract

We investigated the changes in GH-IGF-I axis in non-obese diabetic (NOD)-mice, a model of insulin dependent diabetes mellitus. Diabetic female NOD mice and their age- and sex-matched controls were sacrificed at 4, 14, 21 and 30 days (*30d DM*) after the onset of glycosuria. Serum GH levels increased and serum IGF-I levels decreased in the 30d DM group (182 ± 32% and 45 ± 24% of age-matched controls respectively, *p* < 0.05). Another group (*30d DM + I*) was given SC insulin, and its serum IGF-I levels remained decreased. Liver GH receptor (GHR) and GH binding protein (GHBP) mRNA levels, as well as liver membrane GH binding assays were deeply decreased in the 30d DM group in comparison to controls. GHR message and binding capacity remained decreased in the 30d DM + I group. Renal GHR mRNA was decreased at 21d DM but not at 14d DM, whereas GHBP mRNA remained unchanged throughout the experiment. In conclusion, increased serum GH levels are documented in NOD diabetic mice, similarly to the changes described in humans. The decrease in GHR levels and decreased serum IGF-I in spite of increased circulating GH suggest a state of GH resistance.


Int. Jnl. Experimental Diab. Res., Vol. 1, pp. 9-18
Reprints available directly from the publisher
Photocopying permitted by license only

© 2000 OPA (Overseas Publishers Association) N.V.
Published by license under
the Harwood Academic Publishers imprint,
part of The Gordon and Breach Publishing Group.
Printed in Malaysia.

### Changes in the Growth Hormone-IGF-I Axis in Non-obese Diabetic Mice

DANIEL LANDAU[a,*], YAEL SEGEV[a], RINA ESHET[b], ALLAN FLYVBJERG[c] and MOSHE PHILLIP[a,b]

[a] *Molecular Endocrinology Laboratory, Soroka Medical Center, Ben Gurion University of the Negev, Beer Sheva;*
[b] *Felsenstein Medical Research Center, Institute for Endocrinology and Diabetes, Schneider Children's Medical Center;*
[c] *Institute of Experimental Clinical Research, Aarhus Kommunehospital, Aarhus C., Denmark*

*(Received 25 July 1999; In final form 2 August 1999)*

**We investigated the changes in GH-IGF-I axis in non-obese diabetic (NOD)-mice, a model of insulin-dependent diabetes mellitus. Diabetic female NOD mice and their age- and sex-matched controls were sacrificed at 4, 14, 21 and 30 days (*30d DM*) after the onset of glycosuria. Serum GH levels increased and serum IGF-I levels decreased in the 30d DM group (182 ± 32% and 45 ± 24% of age-matched controls respectively, $p < 0.05$). Another group (*30d DM + I*) was given SC insulin, and its serum IGF-I levels remained decreased. Liver GH receptor (GHR) and GH binding protein (GHBP) mRNA levels, as well as liver membrane GH binding assays were deeply decreased in the 30d DM group in comparison to controls. GHR message and binding capacity remained decreased in the 30d DM + I group. Renal GHR mRNA was decreased at 21d DM but not at 14d DM, whereas GHBP mRNA remained unchanged throughout the experiment. In conclusion, increased serum GH levels are documented in NOD diabetic mice, similarly to the changes described in humans. The decrease in GHR levels and decreased serum IGF-I in spite of increased circulating GH suggest a state of GH resistance.**

*Keywords:* Non-obese diabetic mouse, growth hormone, growth hormone receptor, insulin-like growth factor I

### INTRODUCTION

IDDM is accompanied by long-term complications, including nephropathy, retinopathy, neuropathy and advanced atherosclerosis (Deckert *et al.*, 1978). The appearance of these complications, mainly nephropathy, increases the relative mortality of IDDM patients by 100 times that of the background population (Borch-Johnsen *et al.*, 1985). Growth hormone (GH) and insulin-like growth factors (IGFs) may participate in the development of diabetic kidney disease (Flyvbjerg *et al.*, 1992, 1989; Bach and Jerums, 1990; Phillip *et al.*, 1994; Werner *et al.*, 1990), as well as other diabetic complications (Holly *et al.*, 1988; Houssay, 1936).

*Corresponding author. Department of Pediatrics, Soroka Medical Center. P. O. Box 151, Beer Sheva 84101, Israel. Tel.: 972-7-6403219, Fax: 972-7-6400528, e-mail: ldaniel@bgumail.bgu.ac.il

The classical endocrine effect of pituitary-secreted GH is the induction of liver IGF-I production and secretion. This effect is mediated *via* activation of the specific GH receptor (GHR), which is extensively distributed in many tissues (Chin *et al.*, 1992). Its binding to GH activates a signal transduction pathway (Cunningham *et al.*, 1991), which leads to increased transcription of several early response genes (Gronowski and Rotwein, 1994). Therefore it is possible that GH exerts effects on target tissues which are independent of IGF-I. In addition, diabetic children and adolescents may not grow well (Murata *et al.*, 1994; Phillips and Orawski, 1977). This growth retardation may be due to GH resistance (English *et al.*, 1993).

Experimental diabetes in rats using streptozotocin (STZ) is characterized by suppressed circulating levels of GH (Tannenbaum, 1981). This is contrary to what has been described in humans (Blankestijn *et al.*, 1993; Press *et al.*, 1984; Hayford *et al.*, 1980). In addition, typical diabetic glomerulosclerosis associated with increasing azotemia does not develop, even in long-term follow-up models. STZ may also induce tumoral growth in the kidney, and $\beta$-cell regeneration may appear (Horton *et al.*, 1997; Chieco *et al.*, 1993). In contrast, the non-obese diabetic (NOD)-mouse model may resemble human diabetic nephropathy more closely (Velasquez *et al.*, 1990). The NOD-mice develop hypoinsulinemia secondary to autoimmune destruction of pancreatic $\beta$-cells at an age of between 100 and 200 days (Tochino, 1984), in association with insulitis and autoantibody production (Wicker *et al.*, 1986). As in humans, NOD mice develop proteinuria and significant glomerular lesions, including a prompt increase in glomerular surface area and an increase in mesangial sclerosis (Doi *et al.*, 1990).

No data are available on serum GH levels in NOD mice and their influence on liver IGF-I secretion. In the present study we evaluated the changes in serum GH and IGF-I, and analyzed the expression and activity of liver and kidney GHR and GHBP in NOD mice, up to four weeks after the appearance of diabetes.

### MATERIALS AND METHODS

### Animals

Twelve-week-old female NOD/Alt mice were purchased from Jackson Laboratories (Bar Harbor, ME, USA). Animal breeding complied with the NIH Guide for the Care and Use of Laboratory Animals. The cumulative incidence of overt diabetes in these animals is over 50% in females by 100 days. Animals were housed in standard laboratory cages and fed *ad libitum* normal mouse chow. Animals had free access to unlimited supplies of tap water. The appearance and persistence of glycosuria determined the onset of diabetes. This was checked weekly in all animals using chemstrips (Ketostix, Bayer-Ames, UK). When the urine glucose test was positive, tail capillary blood samples were checked with Glucometer Elite (Bayer Diagnostics, Puteaux Cedex, France). The normal range (99% confidence level) of blood glucose levels for NOD mice is from 3.0 to 9.9 mmol/L. Diabetes was diagnosed when blood glucose levels were above the normal range on two consecutive days. The day that glycosuria was first noted was considered as day 1 of diabetes. Diabetic mice were sacrificed 4 (group *4d DM*), 14 (*14d DM*), 21 (*21d DM*) and 30 (*30d DM*) days after the onset of diabetes. Normoglycemic (as determined by normal blood glucose levels at the day of sacrifice) age-matched female NOD mice were used as controls (group C). Another group of diabetic NOD mice was treated with SC injections of Ultralente human insulin (Novo Nordisk, Denmark) on an every other day basis from the beginning of glycosuria. Insulin dosage was gradually increased to 4 units per injection, which only kept serum glucose levels below 25 mmol/L

(450 mg/dl) and animals non-ketotic. This group was sacrificed after 30 days of diabetes (group *30d DM + I*). Mice were sacrificed by decapitation. Trunk blood was collected and serum was separated and frozen in −20°C to measure later GH and IGF-I levels. Liver and kidney tissue were removed carefully and immediately frozen in liquid nitrogen and then transferred to −70°C.

### Determination of Serum IGF-I

Serum IGF-I was measured after extraction with acid-methanol (30 µl serum and 750 µl acid-methanol). The mixture was incubated for 2 hours at room temperature, centrifuged and 25 µl of the supernatant was diluted 1:200 before analysis. Serum IGF-I was measured by radioimmunoassay (RIA) using a polyclonal rabbit antibody (Nichols Institute Diagnostics, San Capistrano, Calif., USA) and recombinant human IGF-I as standard (Amersham International, Amersham, Bucks, UK). Mono-iodinated IGF-I ($^{125}$I-(tyr$^{31}$)-IGF-I) was obtained from Novo-Nordisk A/S, Bagsvaerd, Denmark. When exposing the serum extract to Western ligand blot (WLB), no IGFBPs could be identified. Furthermore, semilog linearity of biosynthetic IGF-I and serum extracts was seen, indicating antigen similarity and that no IGFBPs interfered in the RIA. Intra- and interassay variability was below 5 and 10%, respectively.

### Serum GH Determination

Serum GH was measured by radioimmunoassay (RIA) using specific polyclonal rabbit anti-rat-GH (rGH) antibody and rGH as standard. Semilog linearity of mouse serum and rGH (in the standard) was found at multiple dilutions, indicating antigen similarity between mouse GH and rGH. The ingredients including $^{125}$I-rGH were obtained from Amersham (Amersham International, Bucks, UK). Intra- and interassay coefficients of variation were less than 5% and 10%. However, in the present study all samples were run in one assay.

### mRNA Analysis

Frozen tissues in 4M guanidinium isothiocyanate were loaded on cesium chloride and total RNA was isolated following the methods as described elsewhere (Phillip *et al.*, 1994). The precipitated RNA was resuspended in sterile $H_2O$ and quantitated by absorbency at 260 nm. The integrity equivalent loading of total RNA was assessed by visual inspection of the ethidium bromide-stained 28S and 18S RNA bands after electrophoresis through 1.25%/2.2M formaldehyde gels. Liver and kidney GHR and GHBP mRNA levels were determined using Northern blot analysis. Twenty µg of total RNA were electrophoresed on 1.3% agarose/2.2M formaldehyde gel in 3-MOPS buffer. The RNA was then transferred onto MagnaGraph (MSI, Westboro, MA, USA) nylon membrane and was crosslinked to the membrane with a UV crosslinker (Hoefer Scientific Instr., San Francisco, CA, USA). A 4.4 kb transcript encoding the GHR and a 1.2 kb transcript encoding the GHBP were detected using a 964 bp fragment of the GHR cDNA, comprising the extracellular domain, the putative transmembrane region, and the short section of the intracellular domain (a gift of L. Mathews). This construct was linearized using BamH1 and radiolabelled with $\alpha$-$^{32}$PdCTP (3000 Ci/mmol; Amersham) by a random primed DNA labelling kit (Boerhinger Manheim, GmbH, Germany). RNA hybridization was performed in a hybridization oven (Micro-4 Hybaid Ltd, UK) at 65°C for 20 hours in hybridization solution (0.2 mM $Na_2HPO_4$, pH 7.2; 7% vol./vol. SDS; 1% wt/vol. BSA; and 1mM EDTA). The washings were done in 0.4X SSC and 0.1% SDS at 65°C. Gels were exposed to Kodak X-Omat AR film (Eastern Kodak, Rochester, NY, USA) at −70°C with 2 intensifying screens. The autoradiograms were quantitated using a phosphorimager (Imagequant, Molecular Dynamics, Sunnyvale, CA, USA).

### Receptor Preparation

Receptor preparation was carried out as previously described (Tushina and Friesen, 1973). Liver was homogenized in 0.25% sucrose solution, centrifuged at 10,000 g for 10 minutes followed by further centrifugation of the supernatant at 100,000 g for 90 minutes, which yielded the microsomal pellet. The pellet was then resuspended in 25 mM Tris-HCl/10 mM $MgCl_2$ buffer (pH 7.4). The quantity of microsomal proteins was measured by the method of Lowry *et al.* (1951), using human serum albumin as a standard. The microsomal fraction was frozen and thawed 3 times and then kept frozen until used for the binding studies (Bergeron *et al.*, 1978). The human somatotropin (hGH, 20 I.U./mg protein) used for iodination and as standard was generously donated by Kabi Diagnostica, Stockholm, Sweden. Na $^{125}I$ (NEZ-033A), carrier free, was purchased from NEN Life Science Products (Boston, USA). Iodination of hGH and binding assays were performed as previously described by us (Eshet *et al.*, 1985). Iodinated preparations were used no more than 2 weeks after iodination. One hundred µl of labelled hGH (25,000 cpm) were incubated with the liver microsomal pellet fraction and 100 µl of various concentrations of unlabelled hGH in 25 mM Tris-HCl/10 mM $MgCl_2$ buffer (pH 7.4) containing 0.1% bovine serum albumin in a final volume of 0.5 ml. This was incubated at 4°C with constant shaking for 48 hours and the incubation was terminated by adding 2 ml ice-cold 0.1% bovine serum albumin/Tris/Mg buffer as noted above. Receptor-bound and free radioactivity was separated by centrifugation at 2000 g for 30 minutes at 4°C. Radioactivity was measured in an autogamma scintillation spectrometer. Parallel incubations were made in the presence of excess (5 µg/ml) unlabelled hormone. Specific binding is the difference between radioactivity bound in the absence (TB, total binding) and the presence (NSB, non-specific binding) of excess unlabelled hormone and is expressed as a percentage of the total radioactivity in the incubation. The capacity and the dissociation constant of liver GH receptor were evaluated using the Scatchard plot analysis.

### Statistical Analysis

Statistical differences between groups were determined by one-way ANOVA for non-repeated measures. Dunnett's test was used to find the source of significance in relation to controls. *P* values equal or less than 0.05 were considered significant. Values are expressed as mean ± SEM unless otherwise stated.

### RESULTS

The mice used in this study ($n = 6$ in each group) became diabetic at a mean age of 104 ± 15 days. Control mice had a mean age of 119 ± 6 days when sacrificed ($p = \text{NS}$). Diabetic mice weight was lower than controls (23 ± 0.6 and 18.3 ± 0.8 grams in the 4d DM and 30d DM groups respectively, *vs.* 25.3 ± 0.7 grams in control animals; $P < 0.01$). The weight of the 30d DM + I group (diabetic animals treated with insulin) was also lower than controls (85.6 ± 2% of control, $P < 0.01$) but higher than the 30d DM animals (21.7 ± 0.61 *vs.* 18.3 ± 0.82 grams; $P < 0.01$) (Fig. 1a).

Serum IGF-I levels did not change during the first two weeks of diabetes, but a significant decrease was observed after 4 weeks in diabetic animals (158 ± 38 *vs.* 351 ± 15 ng/ml in controls, $p < 0.05$). Serum IGF-I remained low in the 30d DM + I group (40 ± 9.9% of controls, $p < 0.05$) (Fig. 1b). Serum GH levels were elevated in the 30d DM group in comparison to controls (51 ± 9 *vs.* 28 ± 2 ng/ml respectively; $p < 0.01$) (Fig. 1). In the 30d DM + I such elevation was

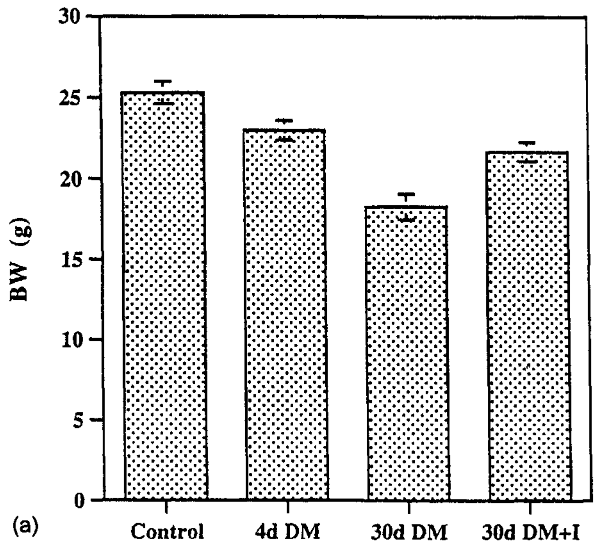

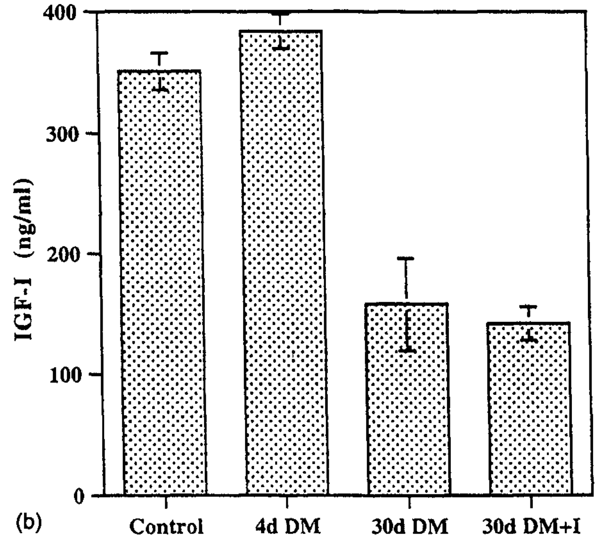

FIGURE 1 (a) Animal weight (BW, in grams), (b) serum IGF-I (in ng/ml) and (c) serum GH (in ng/ml) levels in NOD mice, in controls, 4 and 30 days diabetic (DM) mice. 30DM + I: diabetic mice treated for 30 days with 4 IU of ultralente insulin every other day. *$p < 0.01$ *vs.* control.

not observed (Fig. 1c). Data on serum IGF-I levels have been previously described by us (Segev *et al.*, 1997).

Steady state liver GHR mRNA levels were decreased in diabetic animals already at 4 days (47 ± 10% of control, $p = 0.004$) and persisted over one month of diabetes (33 ± 9% of

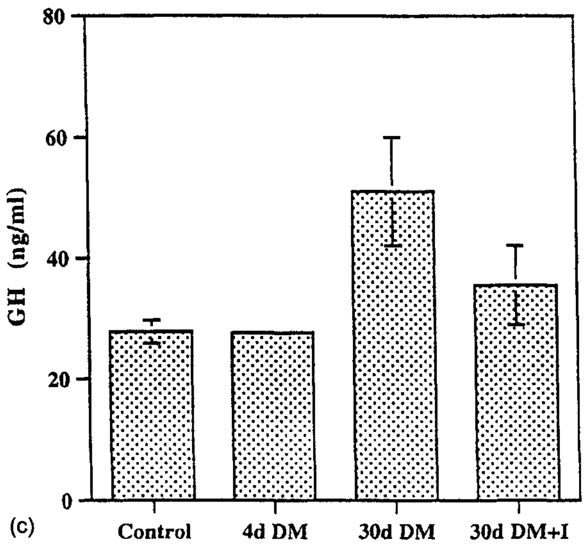

FIGURE 1 (Continued).

age-matched control, $p < 0.001$). The GHR message remained decreased in the 30d DM + I group: mRNA levels remained as low as 20 ± 2% of non-diabetic animals ($p < 0.0001$). Liver GHBP mRNA followed the same pattern as GHR mRNA, and its levels decreased to 67 ± 4% and 30 ± 4% of controls at 4 and 30 days of diabetes respectively ($p < 0.001$) (Fig. 2).

Renal GHR and GHBP mRNA levels showed complex changes in 2 and 3-week non-treated diabetic mice in comparison to controls (Fig. 3). GHBP mRNA remained unchanged in these two time points. Renal GHR mRNA was not significantly decreased at two weeks of DM (77 ± 5.4% of age-matched controls; $p = \text{NS}$), but the receptor message was significantly decreased at 3 weeks of DM (30 ± 10% of control; $p < 0.01$).

GHR binding assays performed on liver membranes demonstrated a linear configuration. We found a decrease in the binding capacity of the receptor in diabetic mice after one month of diabetes in comparison to controls (118 ± 26 *vs.* 475 ± 63 fmol receptor/μg protein; $p < 0.0001$) whereas receptor affinity remained unchanged (0.42 ± 0.12 *vs.* 0.28 ± 0.03 nmol/L;

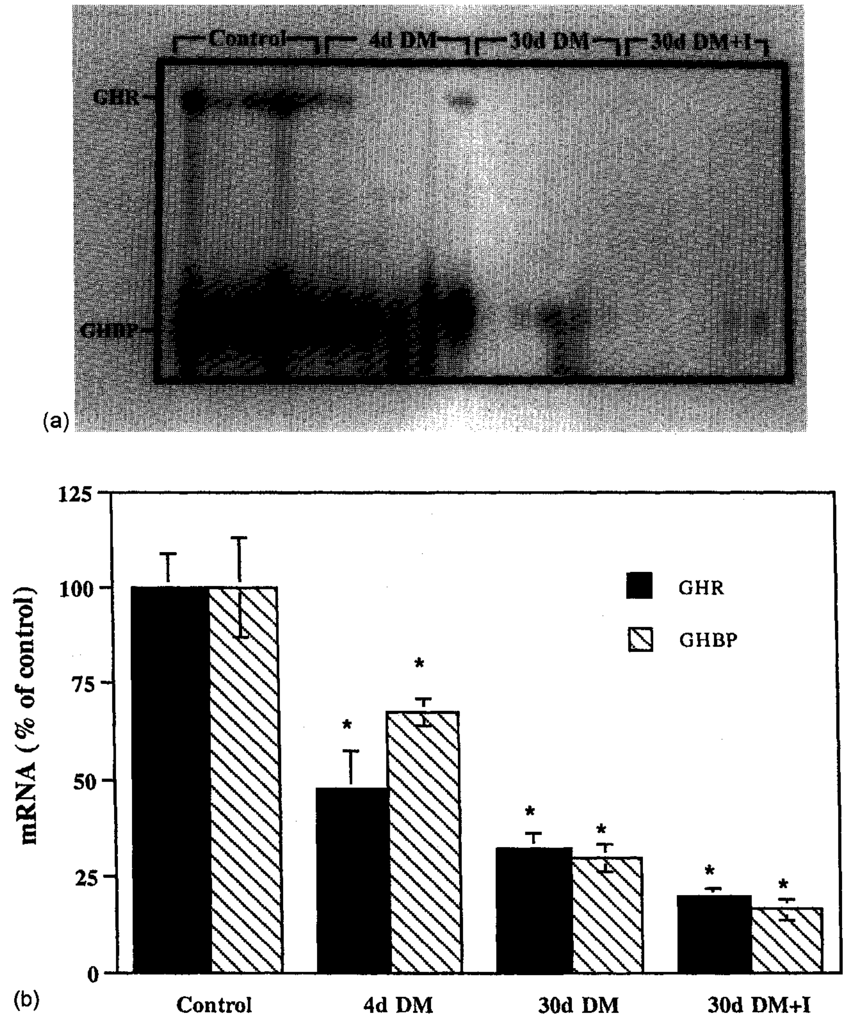

FIGURE 2 (a) Northern blot analysis of liver (20 μg) total RNA of control, 4 days and 30 days diabetic (DM) NOD mice. DM + I: 30 day diabetic animals treated with 4 IU of ultralente insulin. The 4.4 kb and 1.2 kb bands corresponding to the GHR and GHBP are shown on the left. Film was exposed for 96 hours at −70°C with two intensifying screens. (b) Quantitation of liver GHR and GHBP levels from the autoradigraph shown in Figure 2a, using a phosphorimager. The data are expressed as the percentage of age-matched non-diabetic controls. $n = 5$ rats in each group. $^*p < 0.05$ *vs.* control.

$p =$ NS). GHR binding capacity remained low and unchanged in the 30d DM + I group (Fig. 4).

### DISCUSSION

This study shows new evidence on the similarity of the NOD model to human IDDM: as in humans, increased circulating GH levels are found in these hyperglycemic mice, a change that is not modulated by insulin therapy. GH is usually secreted in a pulsatile fashion, and a single measurement of the serum GH may not properly reflect GH levels. However, in the control group GH has also been sampled in the same way. In addition, the decapitation procedure for blood sampling in our experiment has been shown to be a significant stimulus for GH secretion (Takahashi *et al.*, 1971). Thus, the results presented by us reflect a true increase

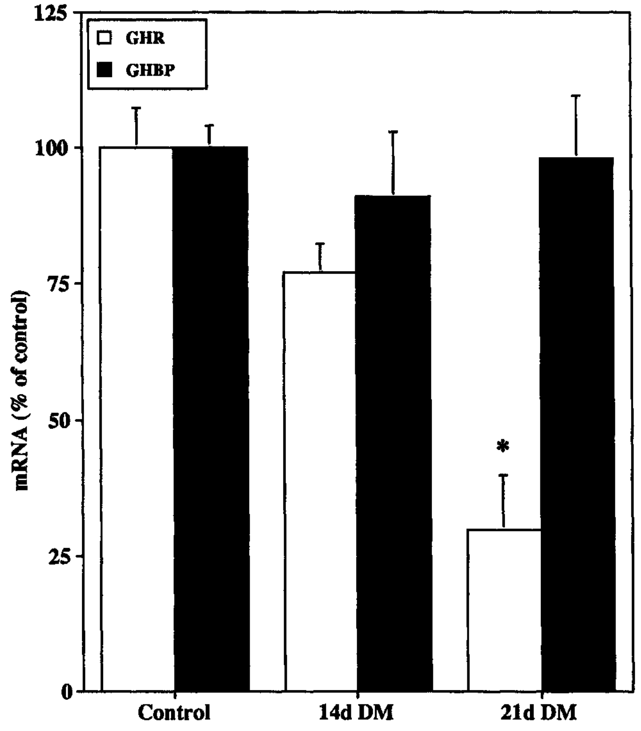

FIGURE 3 Northern blot analysis of kidney GHR and GHBP: 20 μg total RNA of control, 14 and 21 day diabetic (DM) mice were analyzed. Renal GHR and GHBP levels were quantitated using a phosphorimager. Data presented summarize 3 series of experiments, repeated twice each. The data are expressed as the percentage of age-matched non-diabetic controls. $^*p < 0.001$ *vs.* control.

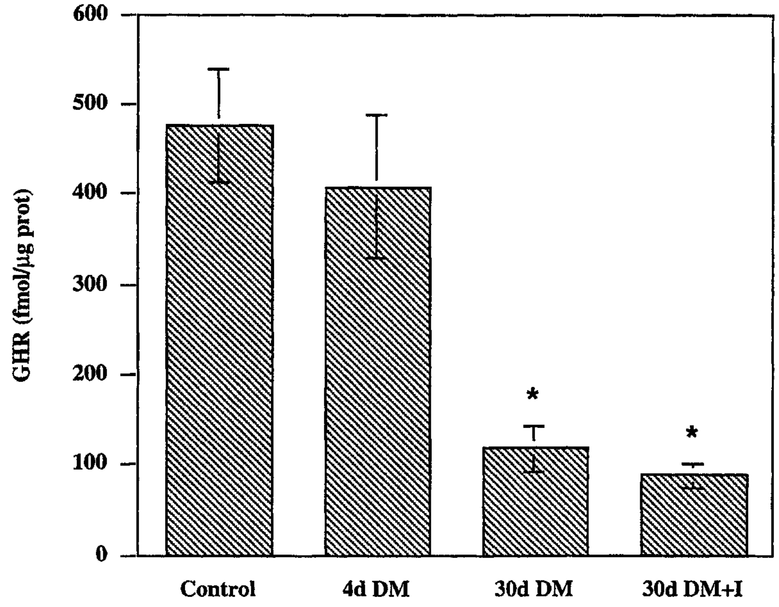

FIGURE 4 Binding capacity of GH receptor sites in liver of NOD non-diabetic (control), in 4 days and 30 days diabetic (DM) NOD mice and in 30 day diabetic animals treated with 4 IU of ultralente insulin (DM+I). $n=6$ in each group. $^*p < 0.0001$.

in serum GH levels in NOD diabetic mice. This is in contrary to previous reports on the usual model of animal IDDM, *i.e.,* streptozotocin injection. In this model decreased circulating GH levels have been described, probably due to inhibition of GH release from the pituitary (Tannenbaum, 1981).

Increased GH action on target tissues may be an important risk factor (independent of IGF-I) for the development of diabetic complications, such as nephropathy and retinopathy. Whereas transgenic mice for bovine-GH develop advanced glomerulosclerosis and die of renal failure, transgenic mice for a variant of GH which antagonizes native GH effects (and causes decreased somatic growth) are protected against the development of nephropathy (Esposito *et al.*, 1996). Recently Doublier *et al.* (1997) provided an additional evidence for the importance of a high GH-low IGF-I environment for the development of glomerulosclerosis: transgenic mice for IGFBP-1 that display such a hormonal combination develop glomerulosclerosis. Doi *et al.* (1990) have shown the natural history of long-standing diabetes in NOD mice. In these animals serum creatinine levels remain normal even after 3 months of diabetes. However, they develop progressive albuminuria and glomerular changes (such as mesangial expansion and sclerosis) that are more similar to diabetic glomerulosclerosis. In the present study we show that renal GHR mRNA decreases only after 3 weeks of diabetes, whereas GHBP mRNA levels remain unchanged. On the other hand, liver GHR and GHBP mRNA are profoundly decreased early after diabetes onset. GHRs are upregulated in diabetic adipocytes, causing an enhanced sensitivity to GH (Solomon *et al.*, 1990). Thus, GHR and GHBP may be differentially regulated in IDDM.

Renal GH binding to kidney tissue is increased in diabetic rats (Marshall *et al.*, 1991). In addition, renal GHBP is upregulated in a model of long-term diabetes (Landau *et al.*, 1998). No change in renal GHR mRNA levels was seen in that model. Kidney IGF-I protein is accumulated in diabetic animals (Segev *et al.*, 1997) together with an increase in kidney IGFBP-1. Given the increased circulating GH levels seen in this model as well as in human disease, an increased biological effect of GH on the kidney tissue (including sclerosis) is possible. However, this hypothesis will have to be further investigated in future studies.

Stunted growth in children with IDDM is a known clinical observation, even though increased GH levels are known in human disease. Since most of the growth promoting actions of GH are mediated by (predominantly liver originated) IGF-I action on bone, a GH insensitivity would be a possible explanation. Such insensitivity to GH could take place at the receptor levels (Bornfelt *et al.*, 1989) or in post-receptor mechanisms (Maes *et al.*, 1986). We show in this study that circulating IGF-I levels are decreased in diabetic mice. In a previous study (Segev *et al.*, 1997) we have shown that this decrease in serum IGF-I is associated with complex changes in the IGFBPs: IGFBP-3 (the predominant carrier of IGF in the circulation) and IGFBP-4 decreased whereas IGFBP-1 increased. Analysis of GH binding to liver membranes also showed a decreased receptor binding capacity, with no change in affinity. Previous studies have shown a decrease in liver GHBP in diabetic states. Plasma concentrations of GHBP are reduced in untreated spontaneously diabetic BB rats, with full normalization during insulin administration (Massa *et al.*, 1993). A decrease in serum GHBP was found in human patients with IDDM (Mercado *et al.*, 1992). Such reduced IGF-I secretion is associated with low hepatic GHR levels that also normalize during insulin treatment (Postel Vinay *et al.*, 1982).

A similar relationship between serum insulin levels and serum GHBP was also shown in humans (Kratzsch *et al.*, 1996). We show here that elevated serum GH and decreased serum IGF-I levels are associated with a decreased expression of liver GHR and GHBP mRNA and GH binding. Insulin treatment did not fully reverse these findings. Previous studies in rats have raised the possibility of a cross binding of hGH with the rat prolactin receptor (Postel Vinay and Desbuquois, 1977). No data is available on hGH binding to prolactin receptor in mice. In addition, our mRNA results support our hypothesis that there is really a decrease in GHR message in IDDM. Even though a significant reduction in serum glucose was achieved in our insulin-treated diabetic animals, no tight control of the hyperglycemic state was reached. Thus, it is possible that a much tighter control of hyperglycemia would cause a reversal of the changes in serum IGF-I and liver GHR expression.

In summary, increased serum GH levels are documented in NOD diabetic mice 4 weeks after the appearance of glycosuria, similarly to the changes described in humans. The decrease in liver GHR levels and decreased serum IGF-I suggest a state of GH resistance at this organ level. This increase in circulating GH may affect target organs for diabetic complications, such as the kidney. Further studies are needed in order to ascertain whether normalizing increased GH levels can modulate those changes.

### *Acknowledgments*

This study was supported by the Sarah Lea and Jesse Z. Shafer Trust.

### *References*

Bach, L. A. and Jerums, G. (1990) Effect of puberty on initial kidney growth and rise in kidney IGF-I in diabetic rats. *Diabetes*, **39**, 557–562.

Bergeron, J. J. M., Posner, B. I., Josefsberg, Z. and Sikstrom, R. (1978) Intracellular polypeptide hormone receptors. The demonstration for specific binding sites for insulin and human growth hormone in Golgi fractions isolated from the liver of female rats. *J. Biol. Chem.*, **253**, 4058–4066.

Blankestijn, P. J., Derkx, F. H., Birkenhager, J. C., Lamberts, S. W., Mulder, P., Verschoor, L., Schalekamp, M. A. and Weber, R. F. (1993) Glomerular hyperfiltration in insulin dependent diabetes mellitus is correlated with enhanced growth hormone secretion. *J. Clin. Endocrinol. Metab.*, **77**, 498–502.

Borch-Johnsen, K., Andersen, P. K. and Deckert, T. (1985) The effect of proteinuria on relative mortality of type 1 (insulin dependent) diabetes mellitus. *Diabetologia*, **28**, 590–596.

Bornfelt, K. E., Arnqvist, H. J., Enberg, B., Mathews, L. S. and Norstedt, G. (1989) Regulation of IGF-I and growth hormone receptor gene expression by diabetes and nutritional state in rat tissues. *J. Endocrinol.*, **122**, 651–656.

Chieco, P., Venturini, A. P., Barbanti, M. and Romagnoli, E. A. (1993) A quantitative cytochemical study on the pathogenesis of streptozotocin-induced epithelial tumors in rat kidney. *Toxicol. Pathol.*, **21**, 402–408.

Chin, E., Zhou, J. and Bondy, C. (1992) Renal growth hormone gene expression: relationship to renal insulin-like growth factor system. *Endocrinology*, **131**, 3061–3066.

Cunningham, B. C., Ultsch, M., De Vos, A. M., Mulkerrin, M. G., Clauser, K. R. and Wells, J. A. (1991) Dimerization of the extracellular domain of the human growth hormone receptor by a single hormone molecule. *Science*, **254**, 821–825.

Deckert, T., Poulsen, J. E. and Larsen, M. (1978) Prognosis of diabetics with diabetes onset before age of thirty-one. *Diabetologia*, **14**, 363–377.

Doi, T., Hattori, M., Agodoa, Y. C., Sato, T., Yoshida, H., Striker, L. and Striker, G. (1990) Gomerular lesions in non-obese diabetic mouse: Before and after the onset of hyperglycemia. *Lab. Invest.*, **63**, 204–212.

Doublier, S., Fouqueray, B., Seurin, D., Verpont, M. C., Gilbert, T., Striker, L. J., Striker, G. E., Binoux, M. and Baud, L. (1997) Overexpression of human insulin-like growth factor binding protein-1 in transgenic mice: a new model of glomerulosclerosis (Abstr). *J. Am. Soc. Nephrol.*, **8**, A2295.

English, D. E., Barnum, C. L., Russell, S. M. and Nicoll, C. S. (1993) Hypophysectomy partially restores the responsiveness of diabetic rats to growth hormone: further evidence of dissociation between growth responses and serum IGF-I concentration. *Endocrine. J.*, **1**, 73–78.

Eshet, R., Peleg, S., Josefsberg, Z., Fuchs, C., Arnon, R. and Laron, Z. (1985) Some properties of the plasma hGH activity in patients with Laron-type dwarfism determine by a radioreceptor assay using human liver tissue. *Horm. Res.*, **22**, 276–283.

Esposito, C., Liu, Z. H., Striker, G. E., Phillips, C., Chen, N. Y., Chen, W. Y., Kopchick, J. J. and Sriker, L. J. (1996) Inhibition of diabetic nephropathy by a GH antagonist: a molecular analysis. *Kidney Int.*, **50**, 506–514.

Flyvbjerg, A., Frystyk, J., Thorlacius-Ussing, O. and Ørskov, H. (1989) Somatostatin analogue administration prevents increase in kidney somatomedin C and initial renal growth in diabetic and uninephrectomized rats. *Diabetologia*, **32**, 261–265.

Flyvbjerg, A., Kessler, U., Dorka, B., Funk, B., Ørskov, H. and Kiess, W. (1992) Transient increase in renal IGF binding proteins during initial kidney hypertrophy in experimental diabetes in rats. *Diabetologia*, **35**, 589–593.

Gronowski, A. M. and Rotwein, P. (1994) Rapid changes in nuclear protein tyrosine phosphorylation after growth hormone treatment *in vivo*. *J. Biol. Chem.*, **269**, 7874–7878.

Hayford, J. T., Danney, M. M., Hendrix, J. A. and Thompson, R. G. (1980) Integrated concentration of growth hormone in juvenile-onset diabetes. *Diabetes*, **29**, 391–398.

Holly, J. M. P., Amiel, S. A., Sandhu, R. R., Rees, L. H. and Wass, J. A. H. (1988) The role of growth hormone in diabetes mellitus. *J. Endocrinol.*, **118**, 353–364.

Horton, L., Fox, C., Corrin, B. and Sönksen, P. H. (1977) Streptozotocin-induced renal tumors in rats. *Br. J. Cancer*, **36**, 692–699.

Houssay, B. A. (1936) The hypophysis and metabolism. *N. Engl. J. Med.*, **214**, 961–970.

Kratzsch, J., Kellner, K., Zilkens, T., Scmidt-Gayk, H., Selisko, T. and Scholz, G. H. (1996) Growth hormone binding protein related immunoreactivity is regulated by the degree of insulinopenia in diabetes mellitus. *Clin. Endocrinol.*, **44**, 673–678.

Landau, D., Domene', H., Flyvbjerg, A., Grönbaek, H., Roberts, C. T. Jr., Argov, S. and LeRoith, D. (1998) Differential expression of renal growth hormone receptor and its binding protein in experimental diabetes mellitus. *Growth Hormone and IGF Research*, **8**, 39–45.

Lowry, O. H., Rosebrough, N. J., Farr, A. L. and Randall, R. J. (1951) Protein measurement with the Folin phenol reagent. *J. Biol. Chem.*, **74**, 265–275.

Maes, M., Underwood, L. E. and Ketelslegers, J. M. (1986) Low serum somatomedin C in insulin dependent diabetes: Evidence for a postreceptor mechanism. *Endocrinology*, **118**, 377–382.

Marshall, S. M., Flyvbjerg, A., Frystyk, J., Korsgaard, L. and Ørskov, H. (1991) Renal insulin-like growth factor I and GHR binding in experimental diabetes and after unilateral nephrectomy in the rat. *Diabetologia*, **34**, 632–639.

Massa, G., Verhaeghe, J., Vanderschueren-Lodeweyckx, M. and Bouillon, R. (1993) Normalization of decreased plasma concentrations of growth hormone binding protein by insulin treatment in spontaneously diabetic BB rats. *Horm. Metabol. Res.*, **25**, 325–326.

Mercado, M., Molitch, M. E. and Bauman, G. (1992) Low plasma growth hormone binding protein in IDDM. *Diabetes*, **41**, 605–609.

Murata, A., Yasuda, T. and Niimi, H. (1994) Two cases of insulin-dependent diabetes mellitus under insulin treatment with slow height velocity: relationship of growth hormone-binding protein, metabolic control and growth. *Acta. Paediatr. Jpn.*, **36**, 272–275.

Phillip, M., Werner, H., Palese, T., Kowarski, A. A. and Stene, M. (1994) Differential accumulation of insulin-like growth factor-I in kidneys of pre- and postpubertal streptozotocin-diabetic rats. *J. Mol. Endocrinol.*, **12**, 215–224.

Phillips, L. S. and Orawski, A. J. (1977) Nutrition and Somatomedin. III. Diabetic control, somatomedin and growth in rats. *Diabetes*, **26**, 864–869.

Postel-Vinay, M. C. and Desbuquois, B. (1977) Interaction of human growth hormone with isolated rat liver cells. *Endocrinology*, **100**, 209–215.

Postel Vinay, M. C., Cohen-Tanugi, E. and Charrier, J. (1982) Growth hormone receptors in rat liver membranes: effect of refeeding and correlation with plasma somatomedin activity. *Mol. Cell Endocrinol.*, **28**, 667–670.

Press, M., Tamborlane, W. V. and Sherwin, R. S. (1984) Importance of raised growth hormone levels in mediating the metabolic derangement of diabetes. *New Engl. J. Med.*, **310**, 810–815.

Segev, Y., Landau, D., Marbach, M., Schadeh, N., Flyvbjerg, A. and Phillip, M. (1997) Renal hypertrophy in hyperglycemic nonobese diabetic mice is associated with persistent renal accumulation of insulin-like growth factor (IGF)-I. *J. Am. Soc. Nephrol.*, **8**, 436–444.

Solomon, S. S., Sibley, S. D. and Cunningham, T. M. (1990) Growth hormone receptors are up-regulated in diabetic adipocytes. *Endocrinology*, **127**, 1544–1546.

Takahashi, K., Daughaday, W. H. and Kipnis, D. M. (1971) Regulation of immunoreactive growth hormone secretion in male rats. *Endocrinology*, **88**, 909–917.

Tannenbaum, G. H. (1981) Growth hormone secretory dynamics in streptozotocin diabetes: evidence of a role for endogenous circulating somatostatin. *Endocrinology*, **108**, 76–82.

Tochino, Y. (1984) Breeding and characteristics of a spontaneously diabetic nonobese strain (NOD mouse) of mice. In: Shafrir, E. and Renold, A. E. (Eds.), *Lessons from Animal Diabetes* (London: John Libbey), pp. 93–100.

Tushina, T. and Friesen, H. G. (1973) Radioreceptor assay for growth hormone. *J. Clin. Endocrinol. Metab.*, **37**, 334–337.

Velasquez, M. T., Kimmel, P. L. and Michaelis, O. E. (1990) Animal models of spontaneous diabetic kidney disease. *FASEB J.*, **4**, 2850–2859.

Werner, H., Shen Orr, Z., Stannard, B., Burguera, B., Roberts, C. T. Jr., and LeRoith, D. (1990) Experimental diabetes increases insulin-like growth factor-I and II receptor concentration in kidney. *Diabetes*, **39**, 1490–1497.

Wicker, L., Miller, B. J. and Mullen, Y. (1986) Transfer of autoimmune diabetes mellitus with splenocytes from the non-obese diabetic (NOD) mice. *Diabetes*, **35**, 855–860.

